# Short-Term Visual Deprivation Does Not Enhance Passive Tactile Spatial Acuity

**DOI:** 10.1371/journal.pone.0025277

**Published:** 2011-09-23

**Authors:** Michael Wong, Erik Hackeman, Caitlin Hurd, Daniel Goldreich

**Affiliations:** 1 McMaster Integrative Neuroscience Discovery and Study, McMaster University, Hamilton, Ontario, Canada; 2 Department of Occupational Therapy, Duquesne University, Pittsburgh, Pennsylvania, United States of America; 3 Department of Psychology, Neuroscience and Behaviour, McMaster University, Hamilton, Ontario, Canada; National Institute of Mental Health, United States of America

## Abstract

An important unresolved question in sensory neuroscience is whether, and if so with what time course, tactile perception is enhanced by visual deprivation. In three experiments involving 158 normally sighted human participants, we assessed whether tactile spatial acuity improves with short-term visual deprivation over periods ranging from under 10 to over 110 minutes. We used an automated, precisely controlled two-interval forced-choice grating orientation task to assess each participant's ability to discern the orientation of square-wave gratings pressed against the stationary index finger pad of the dominant hand. A two-down one-up staircase (Experiment 1) or a Bayesian adaptive procedure (Experiments 2 and 3) was used to determine the groove width of the grating whose orientation each participant could reliably discriminate. The experiments consistently showed that tactile grating orientation discrimination does not improve with short-term visual deprivation. In fact, we found that tactile performance degraded slightly but significantly upon a brief period of visual deprivation (Experiment 1) and did not improve over periods of up to 110 minutes of deprivation (Experiments 2 and 3). The results additionally showed that grating orientation discrimination tends to improve upon repeated testing, and confirmed that women significantly outperform men on the grating orientation task. We conclude that, contrary to two recent reports but consistent with an earlier literature, passive tactile spatial acuity is not enhanced by short-term visual deprivation. Our findings have important theoretical and practical implications. On the theoretical side, the findings set limits on the time course over which neural mechanisms such as crossmodal plasticity may operate to drive sensory changes; on the practical side, the findings suggest that researchers who compare tactile acuity of blind and sighted participants should not blindfold the sighted participants.

## Introduction

Does visual deprivation cause tactile acuity enhancement? This question, important to neuroscientific understanding of tactile perception and of the interaction between the senses, has been investigated for decades.

Early studies reported that tactile perception improved upon prolonged simultaneous deprivation of multiple sensory modalities. Doane and colleagues [1] observed that participants deprived for two days of patterned vision, audition and touch improved in their ability to discriminate one from two points indented into the skin, a finding later confirmed by Nagatsuka and colleagues [2], [3]. Zubek [4] demonstrated that participants deprived for seven days of patterned vision and audition improved in their performance on a tactile fusion task. Participants were presented with successive air jets at progressively increasing frequencies until the stimuli become perceptually fused; fusion at higher frequencies was indicative of better performance.

These findings were soon followed by reports that prolonged visual deprivation alone sufficed to improve tactile perception. Zubek et al. [5], [6] demonstrated that seven days of visual deprivation produced tactile acuity enhancement, as assessed by two-point and tactile fusion tasks; the investigators observed facilitatory effects of visual deprivation when participants were completely light deprived, and also (but to a lesser degree) when participants were deprived of patterned vision.

For a period of several decades following these intriguing early studies, interest in the field seems to have faded. With the advent of functional imaging, interest resurged as many studies revealed that tactile stimuli activate occipital cortical areas in blind participants (crossmodal plasticity) [7]–[14]. Concurrently, perceptual studies revealed heightened tactile acuity in blind compared to sighted participants [15]–[23]. Together, these findings led researchers to hypothesize that visual-deprivation-induced crossmodal plasticity might enable supernormal tactile perception.

It was soon discovered that the occipital cortex of visually deprived sighted participants becomes hyperexcitable [24], [25] and, as observed in blind participants, responsive to tactile inputs [26], [27]. Reexamining the effects of prolonged visual deprivation on the tactile acuity of sighted participants, Kauffman et al. [28] reported that participants' ability to discriminate Braille characters pressed against the passive fingertip improved after five days of visual deprivation, a finding in general agreement with the early literature [1]–[6]. Merabet et al. [27] further showed that transcranial magnetic stimulation applied to the occipital cortex disrupted the ability to distinguish Braille characters among participants who had been blindfolded (and trained on Braille) for five days, but did not affect Braille character discrimination among a control group that had been trained without blindfolding. This result suggested a functional role for the tactile responsiveness acquired by occipital cortex during long-term blindfolding. Neither Kauffman et al. [28] nor Merabet et al. [27] assessed tactile acuity following short-term visual deprivation.

Because tactile responsiveness of occipital cortex occurred within 90 minutes of blindfolding according to one study [26] (but required 5 days of blindfolding according to another [27]), an important unresolved question is whether short-term visual deprivation also results in tactile acuity improvement. The literature on this topic has been controversial. The early literature provided no indication that participants' performance on tactile tasks improved as a consequence of multisensory deprivation spanning two [29], [30], four [31], or eight hours [32], or with eight hours of visual deprivation [33] (see Table 1). However, in some of these early studies the participants were not fully light deprived, but were instead deprived only of patterned vision [31], [32]; furthermore, these early studies used now-outdated assessments, such as two-point discrimination, that have come under serious criticism as invalid measures of tactile spatial acuity [34].

**Table 1:** Summary of visual/multisensory deprivation studies since 1959.

	Deprivation condition				
Study	Vision	Audition	Touch	Deprivation	Task
				Period	
Doane et al. (1959) [1]	Translucent	Mechanical noise	Cotton gloves;	2–3 days	2-point discrimination
	goggles		forearm-length		-index finger
			cardboard cuffs		-forearm
					-upper arm*
					-forehead*
Cohen et al. (1962) [29]	Pitch-dark room	Sound-attenuated	Not deprived	2 hours	2-point discrimination
		room			-palm
					-back of hand
					Letter tracing
					-forehead
					-back of hand
Kamchatnov (1962) [33]	Dark room	Not deprived	Not deprived	8 hours	2-point discrimination
					-index finger
					-thumb
					-upper arm
Pollard et al. (1963) [32]	Translucent dome	White noise	Cotton mittens;	8 hours	2-point discrimination
	or translucent		feet separated &		-test site not specified
	goggles		bound		
Nagatsuka & Maruyama (1963) [2]	Translucent	Semi-soundproof	Cardboard cuffs	2 days	2-point discrimination
	goggles	Room			-back of hand*
Culver et al. (1964) [30]	Pitch-dark room	Sound-attenuated	Not deprived	2 hours	Tactile localization
		room			-palm
Nagatsuka & Suzuki (1964) [3]	Translucent	Semi-soundproof	Cardboard cuffs	2 days	2-point discrimination
	goggles	room			-back of hand*
Reitman & Cleveland (1964) [31]	Translucent	White noise	Cotton gloves;	4 hours	Punctate pressure detection
	goggles		arm-length		-index finger
			cardboard cuffs		-wrist
					2-point discrimination
					-forearm
Zubek (1964) [4]	Translucent	White noise	Heavy leather	7 days	Tactile fusion
	goggles		gloves		-index finger*
					-forearm*
Zubek et al. (1964a) [6]	Black mask	Not deprived	Not deprived	7 days	2-point discrimination
					-palm*
					Tactile fusion
					-index finger*
					-forearm*
Zubek et al. (1964b) [5]	Translucent	Not deprived	Not deprived	7 days	2-point discrimination
	goggles				-palm
					Tactile fusion
					-index finger*
					-forearm*
Kauffman et al. (2002) [28]	Blindfold	Not deprived	Not deprived	5 days	Braille dot discrimination
					-index finger*
Facchini & Aglioti (2003) [35]	Opaque goggles	Not deprived	Not deprived	90 minutes	Grating orientation
					-index finger*
Merabet et al. (2008) [27]	Blindfold	Not deprived	Not deprived	5 days	Punctate pressure detection
					-index finger
					Braille dot discrimination
					-index finger*
					Grating orientation
					-index finger
Leon-Sarmiento et al. (2008) [36]	Opaque goggles	Not deprived	Not deprived	45 minutes	Grating orientation
					-index finger*

*Statistically significant improvement. For a review of the early studies, see Zubek et al. [52].

In contrast to the early literature, two modern studies reported significant effects of short-term visual deprivation on tactile acuity. Comparing a “non-deprived” control group to a visually deprived experimental group, Facchini and Aglioti [35] observed significant tactile acuity improvement upon 90 minutes of visual deprivation. Testing a group of participants first in the light and then upon 45-minutes of visual deprivation, Leon-Sarmiento et al. [36] observed that participants' tactile acuity was significantly better in the second test. Both studies employed the grating orientation task (GOT), a modern gold standard test of passive tactile spatial acuity that is not beset by the limitations of the two-point test [34], [37], [38].

Nevertheless, particular technical aspects of these modern studies may have led the investigators to mistaken conclusions. For instance, Facchini and Aglioti [35] blindfolded all participants for testing; therefore, the performance of their “non-deprived” participants is not necessarily representative of tactile acuity under normal visual conditions. Leon-Sarmiento et al. [36] did not use a counterbalanced design, nor did they perform a post-deprivation test upon the restoration of normal vision, or include a non-deprived control group. In the absence of any of these proper controls it is not possible to know whether their data reflect an effect of visual deprivation or simply a practice effect. Finally, both studies used difficult-to-control manual stimulus delivery, in which the investigator presses the tactile gratings by hand onto the participant's fingertip; unintended manual stimulus variability has the potential to mask differences between conditions or to produce apparent differences where none exist.

Here, we report the results of a study designed to resolve the controversy surrounding the effects of short-term visual deprivation on passive tactile spatial acuity. Ours is the first study of short-term visual deprivation to use a precision-controlled automated tactile grating orientation task [39], and the first to examine the effects of different short-term periods of visual deprivation. In a series of three experiments, we assessed the effects on GOT performance of visual deprivation periods ranging from under 10 to over 110 minutes. The experiments consistently showed that GOT performance does not improve with short-term visual deprivation. We conclude, in agreement with the earlier literature [29]–[33], that passive tactile spatial acuity is resistant to short-term visual deprivation.

## Results

**Figure 1:**
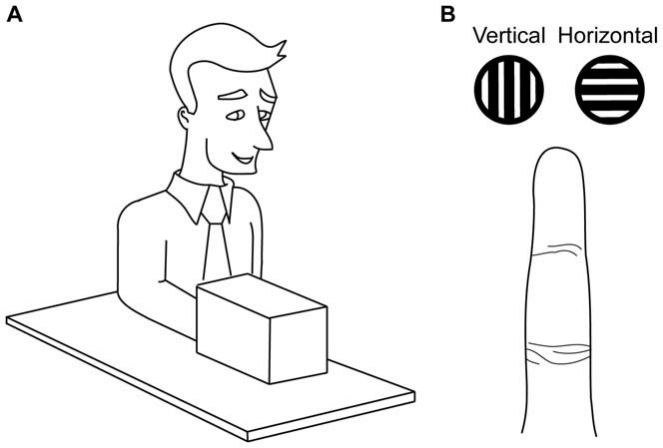
Grating orientation task (GOT). A. Participants were seated upright with their tested hand resting in prone position on a tabletop. In Experiments 2 and 3, a box occluded the participant's tested hand from view. B. In each trial, a grating stimulus contacted the tested finger pad twice, once with the gratings aligned vertically, and once with the gratings aligned horizontally. The images in A and B are not drawn to scale.

In three experiments involving 158 participants, we assessed whether tactile spatial acuity improves with short-term visual deprivation. We tested 48 participants in Experiment 1, 44 participants in Experiment 2, and 66 participants in Experiment 3. We used the GOT, a rigorous test of tactile spatial acuity [34], [37], [38], to assess each participant's ability to discern the orientation of grating stimuli applied to the stationary distal index finger pad of the dominant hand (Figure 1). In all three experiments, we used the Tactile Automated Passive-Finger Stimulator (TAPS), a precision-controlled fully automated tactile stimulus device [39].

### Experiment 1

**Figure 2:**
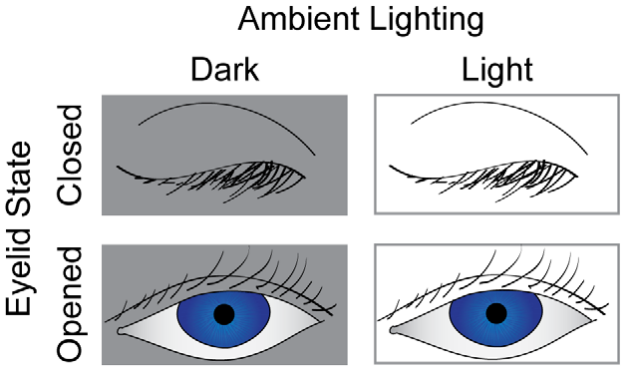
Experiment 1 conditions. In a two-by-two repeated measures design, every participant was tested under four conditions: two conditions of ambient lighting (dark and light) by two conditions of eyelid state (eyes opened and eyes closed). Each participant completed the four conditions twice. The experiment duration was approximately 80 minutes.

To investigate whether tactile spatial acuity improves upon brief periods (e.g., under 10 min) of visual deprivation, we used a 2x2 counterbalanced repeated-measures design, testing 48 sighted participants under all four combinations of ambient lighting (light or pitch-dark) and eyelid state (eyes opened or eyes closed) (Figure 2). After the completion of the four conditions (iteration 1), each participant was tested again on the same four conditions in the same order (iteration 2). Two participants could not complete the majority of the test blocks and were excluded from data analysis.

**Figure 3:**
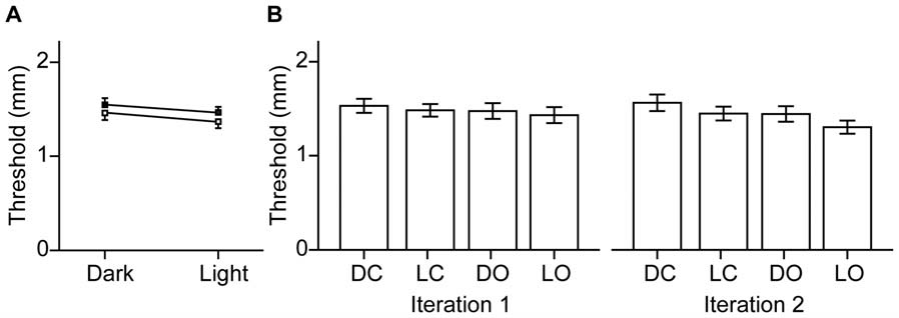
Experiment 1 data. A, Participants' mean 70.71% thresholds are shown for the two conditions of ambient lighting (pitch-darkness, left; indoor fluorescent lighting, right) and eyelid state (eyes closed, filled squares; eyes opened, open squares). The solid lines connecting the symbols illustrate the effect of ambient lighting. Errors bars represent 1 SEM; the error bars on upper and lower symbols are displaced in opposite directions for visual clarity. B, Participants' mean 70.71% thresholds are plotted for each condition in the first and second iterations separately (darkness with eyes closed, DC; light with eyes closed, LC; darkness with eyes opened, DO; light with eyes opened, LO). Data in (A) and (B) are from 46 participants.

To examine the effects of ambient lighting and eyelid state, we performed a 2 (ambient lighting) x 2 (eyelid state) x 2 (iteration) x 2 (sex) ANOVA. This analysis revealed significant main effects of ambient lighting (p = 0.010) and of sex (p = 0.029). Participants' tactile acuity worsened significantly with visual deprivation, and women significantly outperformed men. On average, thresholds in the dark were 0.09 mm higher than in the light (95% confidence interval, 0.02 – 0.15 mm) (Figure 3A), and men's thresholds were 0.25 mm higher than women's (95% confidence interval, 0.03–0.48 mm).

Although the effect of eyelid state was not significant (p = 0.077), participants tended to perform better with eyes opened than closed. The effect of iteration was not significant (p = 0.396), but participants tended to perform better in iteration 2 than iteration 1, suggestive of a practice effect (Figure 3B).

We next examined whether the elevation of tactile threshold in the dark depended upon the dark/light testing order. For each participant we computed a difference score: threshold of first iteration 1 test in the dark – threshold of first iteration 1 test in the light. For instance, for a participant tested in the order LC, DC, DO, LO (see Figure 3 legend for definitions), the difference score was DC threshold minus LC threshold. We compared the differences scores of participants tested initially in the light to those of participants tested initially in the dark. An independent-samples t test revealed no significant difference between groups (p = 0.251), but the mean difference score was considerably larger for participants initially tested in the dark (0.16 mm ±0.11 mm; mean ± SE) than for those initially tested in the light (−0.03 mm ±0.13 mm). We observed the same (non-significant, p = 0.129) trend in the data from iteration 2: the mean difference score (threshold of first iteration 2 test in the dark – threshold of first iteration 2 test in the light) was considerably larger for participants initially tested in the dark (0.20 mm ±0.10) than for those initially tested in the light (−0.02 mm ±0.09). A parsimonious explanation for this order effect is that it is due to the superposition of two underlying effects: while visual deprivation worsens acuity (elevates threshold), practice tends to improve acuity (lower threshold). Thus, for participants tested in the dark then light, the two effects acted in the same direction, producing a large threshold difference; for participants tested in the light then dark, the two effects acted in opposite directions, nullifying the threshold difference.

### Experiment 2

Having observed no improvement in tactile spatial acuity with brief visual deprivation (Experiment 1), we wondered whether a longer period of visual deprivation would improve participants' tactile spatial acuity and, if so, whether the improvement would occur abruptly or gradually. Accordingly, in Experiment 2 we lengthened the visual deprivation period to 70 minutes.

**Figure 4:**
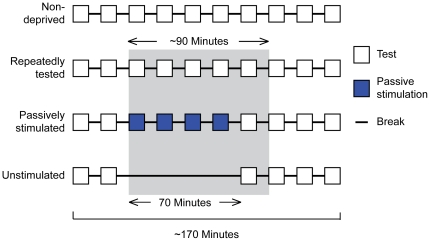
Experiment 2 conditions. One group of non-deprived and three groups of visually deprived participants were tested on the GOT (white squares). In the passively stimulated group, participants received grating stimuli that they were not required to discriminate (blue squares). Blocks were separated by 8-minute rest periods (short horizontal lines). The shaded rectangle indicates the period of visual deprivation. The experiment duration was approximately 170 minutes.

Participants were assigned to one of four groups. In the non-deprived group, participants were tested in the light 10 times. In the three visually deprived groups, participants were tested in the light twice before and three times after a period of 90 minutes in the pitch-dark. The sequence of events in the dark differed by group (Figure 4). We conducted the experiment until each group contained 10 participants who had successfully completed testing. This required the testing of 44 participants in total, because four participants could not perform the task beyond chance level and were therefore excluded from data analysis.

**Figure 5:**
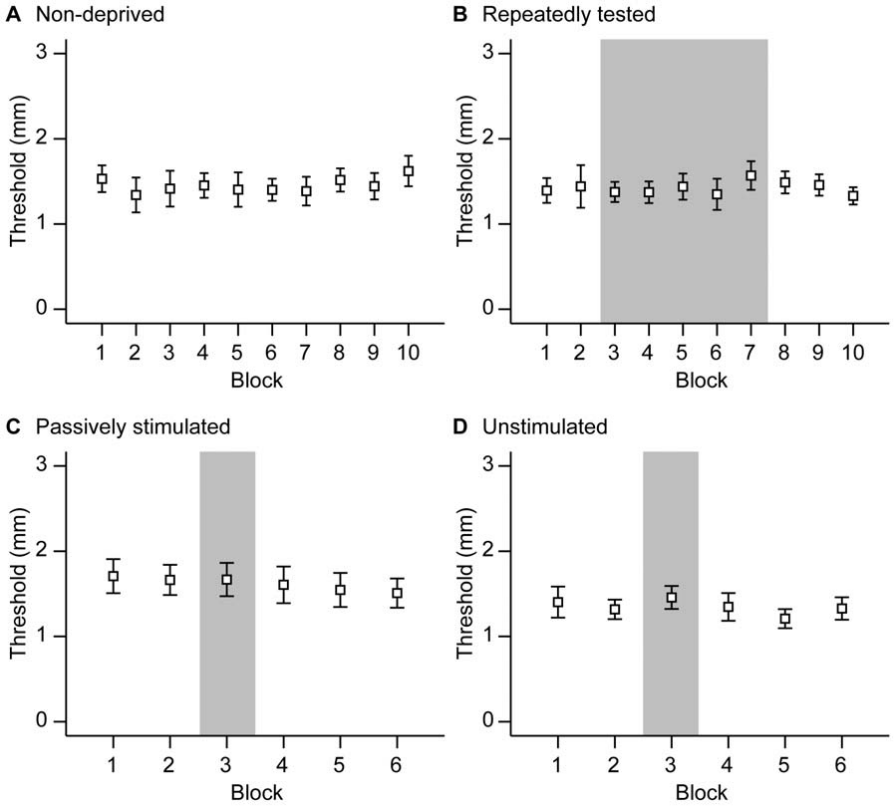
Experiment 2 data. GOT performance (mean 76% threshold) of the four groups (n = 10 participants per group): A, non-deprived. B, repeatedly tested. C, passively stimulated. D, unstimulated. The shaded rectangles in B–D delineate the visual deprivation period. Errors bars represent ±1 SEM.

To analyze the data from each group, we performed a one-way repeated-measures ANOVA across testing blocks. We observed no significant change in GOT performance within any group (Figure 5): non-deprived (10 blocks, p = 0.711), repeatedly tested (10 blocks, p = 0.941), passively stimulated (6 blocks, p = 0.677), unstimulated (6 blocks, p = 0.361). These results indicate both that the participants' performance in the dark was equivalent to their performance in the light, and that performance did not improve significantly with practice. As in Experiment 1, the data suggested a non-significant practice trend (e.g., compare the first and final test block thresholds in Fig. 5B, C, D).

To examine the effect of sex, we averaged the threshold of each participant across all test blocks and performed an independent-samples t test to compare the mean thresholds for women and men. This analysis revealed that women significantly outperformed men (p = 0.015). On average, men's thresholds were 0.35 mm higher than women's (95% confidence interval, 0.07 – 0.63 mm).

### Experiment 3

Having observed no improvement in tactile spatial acuity in Experiments 1 and 2, we wondered whether a somewhat longer period of deprivation might result in acuity enhancement. In addition, we wondered whether participants might have lost alertness during the visual deprivation period in Experiment 2, perhaps resulting in a worsening of performance that masked a true benefit of visual deprivation. Accordingly, we further lengthened the visual deprivation period to 110 minutes, and to safeguard participant alertness we recruited participants in sets of three and encouraged conversation during the visual deprivation period. In keeping with Facchini and Aglioti [35], we decided to use just two groups of participants – a visually deprived group and a non-deprived group – and to test each participant just three times.

**Figure 6:**
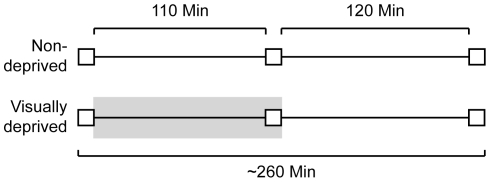
Experiment 3 conditions. A non-deprived group and a visually deprived group of participants were tested three times on the GOT (white squares). A 110-minute conversation period separated the first and second tests, and a 120-minute break separated the second and third tests. The shaded rectangle indicates the period of visual deprivation. The experiment duration was approximately 260 minutes.

We tested 66 participants. Five participants could not perform the task beyond chance level, and were therefore excluded from data analysis. Each set of three participants was assigned to one of two groups: a non-deprived group (n = 29) and a visually deprived group (n = 32). Participants in both groups were tested three times: before a 110-minute conversation period, immediately following the conversation period, and 120 minutes following the second test. Whereas participants in the non-deprived group were always in the light, those in the visually deprived group were in the pitch-dark during the conversation period and the second test (Figure 6).

To examine whether 110 minutes of visual deprivation improves GOT performance, we performed a 3 (test block) x 2 (group: visually deprived, non-deprived) x 2 (sex) ANOVA. This analysis revealed a significant main effect of sex (p = 0.008), indicating that women outperformed men. There was no significant main effect of test block or of group, nor was there a significant test block x group interaction. Thus, visual deprivation did not affect tactile spatial acuity.

**Figure 7:**
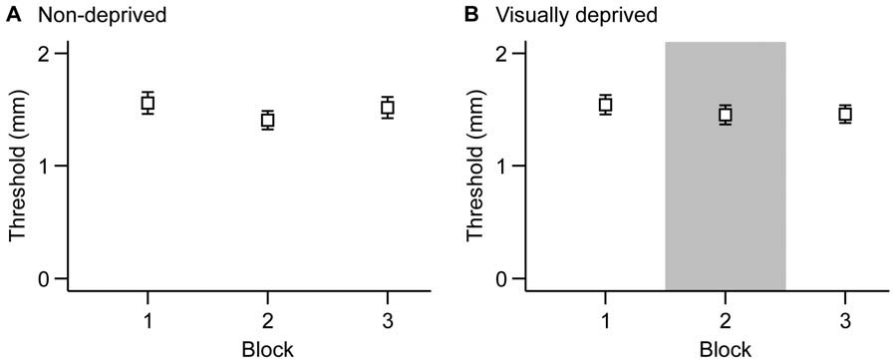
Experiment 3 data. GOT performance (mean 76% threshold) of the two groups: A, non-deprived (n = 29 participants). B, visually deprived (n = 32 participants). The shaded rectangle in B delineates the visual deprivation period. Error bars represent ±1 SEM.

One-way repeated-measures ANOVAs performed separately for each group confirmed that across the three test blocks there was no significant change in the performance of participants in the visually deprived group (p = 0.435) (Figure 7A) or the non-deprived group (p = 0.115) (Figure 7B). As in Experiments 1 and 2, however, we observed a non-significant trend for improvement with repeated testing. In both groups, first test thresholds were greater than second and third test thresholds; from test 1 to test 2, thresholds decreased on average by 0.15 mm in the non-deprived group (Figure 7A) and by 0.09 mm in the visually deprived group (Figure 7B).

To quantify the difference between thresholds of men and women, we averaged each participant's thresholds across the three tests (without regard to group). On average, men's thresholds were 0.28 mm higher than women's (95% confidence interval, 0.08 – 0.48 mm).

## Discussion

Contrary to previous reports [35], [36], we have shown that short-term visual deprivation does not improve tactile spatial acuity as measured with the GOT. Across three experiments, participants' ability to discern grating orientation either worsened slightly or remained stable following visual deprivation.

### Short-term visual deprivation does not enhance tactile spatial acuity

The experiments reported here provide clear and consistent evidence that short-term visual deprivation does not enhance passive tactile spatial acuity.

Using a counterbalanced repeated-measures design, we found in Experiment 1 that tactile spatial acuity actually worsened to a small but significant degree upon short-term visual deprivation. Participants performed significantly worse in the dark than in the light, and tended (although not significantly) to perform worse with their eyes closed than opened. Of the four conditions under which they were tested, participants performed best on average in the condition with the greatest visual stimulation (eyes opened in the light). These results may be attributable to a loss of alertness experienced during visual deprivation, or to some other cause.

Tracking tactile spatial acuity over 70 minutes of visual deprivation, we found in Experiment 2 that this period of deprivation did not result in tactile perceptual improvement. This was true irrespective of whether during visual deprivation the participant received no tactile stimulation, unattended tactile stimulation, or repeated GOT testing. Similarly, in Experiment 3, participants performed equivalently before and after 110 minutes of visual deprivation. Thus, our results consistently show that tactile spatial acuity does not improve during short-term visual deprivation.

In contrast to Experiment 1, in Experiments 2 and 3 we did not observe a significant worsening of GOT performance upon visual deprivation. We propose that this apparent discrepancy is explained by two factors: 1) The fact that all visually-deprived participants in Experiments 2 and 3 were first tested in the light, whereas half the participants in Experiment 1 were first tested in the dark, and 2) The fact that all three experiments revealed a trend (although non-significant) for thresholds to decrease slightly with repeated testing, consistent with previous reports (for non-significant GOT practice effects, see [17], [40]; for significant practice effect, see [41]).

Because of the practice effect trend, participants tested first in the light and then in the dark will tend to show nearly equivalent performance on the two tests: the worsening of acuity due to visual deprivation is counteracted to some degree by the practice effect. This phenomenon seems evident in much of the data from Experiments 2 and 3. For instance, Figures 5C and D (and, to a lesser extent, Fig. 5A) suggest a tendency for thresholds to lower with repeated testing in the light. That practice effect trend, however, appears to be largely arrested (Fig. 5C) or counteracted (Fig. 5D) upon visual deprivation, resuming only upon the return of the participant to the light (Fig. 5B, C, D). Similarly, Figures 7A and B both show a trend for thresholds to lower between blocks 1 and 2, but this trend is slightly smaller in Figure 7B than in Figure 7A, presumably because the effect of visual deprivation partially counteracted the practice-associated threshold reduction.

Consistent with this interpretation, we noticed in Experiment 1 that the mean threshold difference score (first test in the dark – first test in the light) was large and positive only among participants who were initially tested in the dark (0.16 mm). The corresponding difference score for participants who were initially tested in the light (−0.03 mm) indicates that those participants did not on average worsen when subsequently tested in the dark. This trend repeated in iteration 2 (0.20 mm vs. −0.02 mm). A parsimonious explanation for this order effect is that it is due to a trend for practice to improve acuity (lower thresholds) from one testing block to the next, together with a trend for visual deprivation to worsen acuity (raise thresholds).

This explanation reconciles the apparent discrepancy between Experiment 1, which revealed a slight but significant worsening of acuity under conditions of visual deprivation, and Experiments 2 and 3, which did not. Experiment 1 used a counterbalanced design so that the average difference observed between conditions was robust against practice effects, whereas Experiments 2 and 3 always tested participants in the light prior to testing them in the dark.

Most importantly, we note that Experiments 1, 2 and 3 all clearly support the conclusion that short-term visual deprivation does not *improve* tactile spatial acuity. If our explanation above is correct, all three experiments indeed lend support to the conclusion that tactile spatial acuity tends to *worsen* under short-term visual deprivation.

### Comparison to previous visual deprivation GOT studies

Our results stand in stark contrast to those of Facchini and Aglioti [35], who reported that participants' tactile spatial acuity significantly improved after 90 minutes of visual deprivation, and Leon-Sarmiento et al. [36], who reported improvement after just 45 minutes of visual deprivation (each study reported approximately 0.2 mm average reduction in GOT threshold following light deprivation). Although our results disagree with those of Facchini and Aglioti [35] and Leon-Sarmiento et al. [36], they are in general agreement with the results of earlier studies [29]–[33] that reported no effects on tactile acuity of short-term visual or multisensory deprivation. The results from the present study, however, are most directly comparable to those of Facchini and Aglioti [35] and Leon-Sarmiento et al. [36], because unlike the earlier studies, Facchini and Aglioti [35], Leon-Sarmiento et al. [36] and the current study used the GOT to test passive tactile spatial acuity.

How might the results of Facchini and Aglioti [35] and Leon-Sarmiento et al. [36] be understood in light of the results of the present study? It is possible but unlikely that the discrepancy between these studies and ours owes to random statistical fluctuation. Facchini and Aglioti [35] tested 28 participants divided equally into visually deprived and non-deprived groups. Leon-Sarmiento et al. [36] tested 13 neurologically normal participants (for comparison with hyperhidrosis patients). Each of our experiments had sample sizes greater than those of [35], [36]. Given the respectable sample sizes of the three studies, we would expect random statistical fluctuation to produce only minor variation in average threshold values.

If the discrepancy between studies did not arise from statistical fluctuation, another possibility is that it arose from unintended variability in stimulus-delivery parameters. The GOT provides a rigorous measure of tactile spatial acuity by assessing participants' ability to discern the orientation of grating stimuli pressed orthogonally against a body part [34], [37], [38]. However, even small non-orthogonal movement upon contact with the test site greatly facilitates perception of grating orientation. Following common practice, Facchini and Anglioti [35] and Leon-Sarmiento et al. [36] used manual stimulus delivery, the investigator pressing the gratings by hand against the participant's skin. In such cases, avoiding unintended movement and controlling a host of other stimulus-delivery parameters (e.g., contact force, onset velocity, stimulus duration) is very difficult even with great care and concentration on the part of the experimenter. It is for these reasons that we prefer to use a precision-controlled automated testing device to conduct the GOT [39].

Two additional methodological considerations may explain the discrepancy between these studies and ours. First, Leon-Sarmiento et al. [36] tested all subjects initially in the light, and next at the end of a 45-minute period of visual deprivation. Unfortunately, the investigators did not use a counterbalanced design (in which half the participants would have been tested in the opposite order), nor did they include a third test after restoration of the light, or test a non-deprived control group. In the absence of any of these controls it is not possible to know whether the results obtained were due to an effect of visual deprivation, or simply to a practice effect.

Second, Facchini and Aglioti [35] used opaque goggles to blindfold the participants in both groups for testing purposes. Thus, one of their groups (the “visually deprived” group) was continuously blindfolded (during test 1, a 90-minute inter-test interval, and test 2), whereas the other (the “non-deprived” group) was in fact also blindfolded, but only during testing. Perhaps these intermittently blindfolded participants performed poorly on each test, as their attention to the task was distracted by the recent addition of the goggles, whereas the continuously blindfolded participants likewise performed poorly on the first test but then habituated to the goggles over time, returning towards normal performance for the second test. (When later tested blindfolded for a third time, following a prolonged period of light exposure, the performance of both groups would once again worsen towards a similar level, as observed). Unfortunately, Facchini and Aglioti [35] did not test participants un-blindfolded and in the light, either before or after the blindfold tests. In the absence of this crucial comparison condition, it is not possible to know whether the apparent improvement of their continuously blindfolded group was in fact simply a return towards normal performance.

### Practical implications for sensory testing studies

In light of the results of Experiment 1, we caution against the blindfolding of sighted participants in tactile psychophysics studies, as this procedure may inadvertently worsen participants' tactile acuity. For instance, although it is becoming increasingly clear that the tactile acuity of blind participants is better than that of sighted participants [15]–[23], blindfolding sighted participants during testing may exaggerate the extent to which blind participants are better. This may explain the larger mean GOT difference between blind and sighted participants (0.42 mm) reported by Van Boven et al. [22] – who blindfolded their sighted participants – than by Goldreich and Kanics [17] (0.33 mm) and Wong et al. [23] (0.2 mm), who tested their sighted participants un-blindfolded and in the light.

Another practical consequence of this study is that investigators of tactile spatial acuity should be aware of the tendency for women to outperform men, and design and analyze their studies accordingly. In all three experiments reported here, we found that women significantly outperformed men on the GOT. This result is consistent with previous reports [17], [22], [23], [42]. A study from our laboratory [42] revealed that the better acuity of women owes to their smaller fingers, and provided some evidence in support of the hypothesis that Merkel mechanoreceptors are more densely packed within smaller fingers. Thus, we recommend that investigators performing between-groups studies (e.g., comparisons between blind and sighted participants) take care to maintain participant sex ratios equal across groups, and / or to incorporate participant sex – if not finger size - as a factor in their statistical analyses.

### Effects of prolonged visual deprivation and crossmodal plasticity

In contrast to short-term visual deprivation, several studies have reported that prolonged visual deprivation does drive tactile acuity enhancement [5], [6], [27], [28]. Surprisingly, however, Merabet et al. [27] found that five days of visual deprivation coupled with Braille training were insufficient to improve participants' performance on the GOT beyond the levels of improvement observed in a non-visually-deprived Braille-trained control group (a significant effect of visual deprivation was found only on a Braille character recognition task, not on the GOT). Thus, it is possible that the GOT taps into a feature of tactile processing that is particularly resistant to improvement with visual deprivation. Alternatively, it is possible that the multi-day tactile training regimen undertaken by the participants in Merabet et al. [27] resulted in ceiling GOT performance, precluding additional effects of visual deprivation. These possibilities should be investigated in future studies.

What neural mechanism might underlie visual deprivation-induced tactile acuity enhancement?

In the absence of vision, the visual cortex becomes responsive to tactile inputs (crossmodal plasticity) [26], [27]. Tactile activation of primary visual cortex appears to be weak, if present at all, within two hours of visual deprivation [26], [43], and emerges more robustly after five days of deprivation [27]. Correspondingly, the results of the present study and others indicate that tactile acuity is unaffected by short-term (minutes to hours) visual deprivation [29]–[33], but improves upon long-term (days) visual deprivation [5], [6], [27], [28]. These observations raise the hypothesis that crossmodal plasticity underlies the tactile acuity enhancement observed upon prolonged visual deprivation. In support of this hypothesis, transcranial magnetic stimulation (TMS) applied to the occipital cortex of sighted participants who were visually deprived for five days disrupted their ability to perform a Braille character discrimination task on which they had been previously trained [27].

Crossmodal plasticity coupled with extensive daily reliance on the sense of touch may also underlie tactile acuity enhancement in blindness [15]–[23].

### Conclusion

In three experiments, we show consistently that short-term visual deprivation for periods up to 110 minutes does not enhance passive tactile spatial acuity. We note that in contrast to short-term visual deprivation, prolonged visual deprivation does reportedly drive tactile acuity enhancement. Investigations that couple perceptual testing with neural imaging will help to elucidate the mechanism by which prolonged visual deprivation enhances tactile acuity.

## Materials and Methods

We conducted three experiments involving 158 participants. None of the participants tested in one experiment were tested in any other. None of the participants had previous experience with the grating orientation task. Experiment 1 was conducted at Duquesne University (Pittsburgh, PA, USA) and Experiments 2 and 3 at McMaster University (Hamilton, ON, Canada).

### Ethics Statement

Experiment 1 was approved by the Duquesne University Institutional Review Board; Experiments 2 and 3 were approved by the McMaster University Research Ethics Board. All participants provided written consent and received monetary compensation and/or course credit for their participation.

### Experiment 1

#### Participants

Forty-eight normally sighted participants (24 men, 24 women, ages 18.4–22.8 years, median age 20.9 years) took part in Experiment 1. Inclusion criteria ensured that participants did not have (by self report) dyslexia, diabetes, nervous system disorders, or injuries or calluses on the index finger of the dominant hand (the finger was inspected in the laboratory to verify its condition). Dyslexia was an exclusion criterion because it has been shown to adversely affect tactile spatial perception [44]. Diabetes was an exclusion criterion because it can affect peripheral nerve conduction, even when neuropathy is not evident [45]. Hand dominance was assessed by a handedness questionnaire (modified from [46]). A subset of the data collected from these participants (performance in the light-eyes open condition) has been reported previously [42].

#### Psychophysical Procedures

We assessed each participant's ability to discern the orientation of grating stimuli applied to the distal index finger pad of the dominant hand. The stimuli were a set of custom-made square-wave gratings, with groove widths ranging from 0.25 mm to 3.1 mm (in increments of 0.15 mm). We used the Tactile Automated Passive-finger Stimulator (TAPS) to mechanically deliver the grating stimuli; see [39] for a complete description of this computer-controlled device. Briefly, the participant's dominant arm rested on a tabletop in prone position, with the distal index finger pad placed over a small circular opening in the table; the gratings were mechanically driven to rise through this opening to contact the finger pad for approximately 1 s (50 g contact force, 4 cm/s onset velocity). Plastic barriers surrounded the finger to ensure that it remained centered on the opening, and a force sensor on the cuticle detected even minor finger movements; the computer system automatically discarded any trials in which finger movements occurred.

In each two-interval forced-choice (2-IFC) trial, the participant's tested finger pad was contacted twice by the grating stimuli, once with the gratings aligned parallel to the long axis of the finger (vertical), and once with the gratings aligned transverse to the long axis of the finger (horizontal); the presentation order was chosen randomly (i.e., horizontal before vertical, or vertical before horizontal). An interstimulus interval of 2s separated the presentation of the two orientations. The participant indicated, by pressing one of two buttons with the non-tested hand, whether the horizontally aligned gratings contacted the tested finger in the first or second interval (Figure 1). Participants were given auditory feedback for correct and incorrect responses after each trial.

We used a two-down one-up adaptive staircase procedure [47] to estimate the groove width that corresponds to 70.71% correct performance (70.71% threshold) – the dependent measure for this experiment. Each staircase began at a groove width of 1.45 mm; thereafter, the groove width was made incrementally thinner (more difficult to perceive) for every two consecutive correct responses, and incrementally wider (easier to perceive) for each incorrect response. To quickly bracket each participant's 70.71% correct threshold, we used an increment size of 0.3 mm until three reversal points occurred (trials at which the staircase changes direction). To obtain a more precise estimate of each participant's 70.71% threshold, we then reduced the increment size to 0.15 mm and ran the staircase until 11 further reversal points were encountered. We averaged the groove widths of these final 11 reversal points to obtain an estimate of the participant's 70.71% threshold. If the participant responded correctly twice at the thinnest groove width (0.25 mm) or incorrectly once at the widest groove width (3.1 mm), that groove width was used as the participant's last reversal point; we then averaged the groove widths beginning with the fourth reversal point and ending with this last reversal point to obtain an estimate of the participant's 70.71% threshold.

#### Experimental Design & Conditions

We tested every participant twice under all four combinations of ambient lighting (light or pitch-dark) and eyelid state (eyes opened or eyes closed). The order of conditions was counterbalanced across participants, such that each of the 24 men was tested on one of the 24 (i.e., 4 factorial) possible combinations of these four conditions, and similarly for each of the 24 women. After completing the four conditions, the participant was tested again on the same conditions and in the same testing order.

Participants took on average 8 minutes to complete a testing block. The mean elapsed time between the end of one block and the start of the next was 2 minutes for blocks within the same iteration. The mean elapsed time between iterations (end of block 4 of iteration 1 to start of block 1 of iteration 2) was 3 minutes. The experiment duration averaged approximately 80 minutes.

During the light conditions, the participants were tested under fluorescent overhead room lighting typical of a well-lit indoor environment. During the dark conditions, the participants were tested in the pitch-dark. Room darkness was such that no visual input was perceptible, even of large nearby objects (e.g., it was not possible to see one's own hand placed in front of the face). The light intensity was less than 0.01 lux, the lower detection limit of our light meter (Mannix DLM2000). To achieve visual deprivation, we chose here (and in Experiments 2 and 3) to use a pitch-dark room rather than blindfolding the participants. A simple cloth blindfold does not screen out all light, and also rubs and tickles against the eyes and face, causing a tactile distraction. Opaque goggles (such as painted swim goggles) can screen out all light, but require tight fits to the eye sockets, and are consequently both distracting and uncomfortable. We wished to test participants without light, and without inducing discomfort or distraction.

An experimenter remained in the testing room at all times to ensure the participants' compliance with the eyelid state (eyes opened or closed) instructions appropriate to the condition. During the light conditions, the experimenter simply viewed the participant's eyes with unaided vision. During the dark conditions, the experimenter periodically verified that the participant's eyes were opened or closed as per condition with the aid of an infrared night vision monocular (Bushnell). Because the infrared beam cast by the night vision device bled somewhat into the visible red, we secured an opaque occluder with a pinhole cutout over the beam source to reduce the size of the beam to the bare minimum needed to obtain a view of the participant.

### Experiment 2

#### Participants

Forty-four normally sighted right hand-dominant students from McMaster University (14 men, 30 women, ages 20.1–25.75 years, median age 21.1 years) participated in Experiment 2. Hand dominance was confirmed by questionnaire (modified from [46]). Inclusion criteria ensured that participants did not have (by self report) dyslexia, diabetes, nervous system disorders, or injuries or calluses on the index finger of the right hand.

#### Psychophysical Procedures

The TAPS device used in Experiment 1 was again used in Experiment 2 to administer the GOT. Here we programmed TAPS to follow a more sophisticated psychophysical adaptive procedure than that used in Experiment 1, a modified version of the Bayesian adaptive ψ-method [39], [48], to estimate each participant's 76% correct threshold – the dependent measure used in this experiment. We implemented a “Bayesian guessing factor” (described in detail in [23], [39]) to assess whether each participant was capable of performing the GOT. Those deemed to be guessing by the Bayesian guessing factor were excluded from data analysis.

Before finger testing commenced, participants were familiarized with the GOT by completing 20 practice trials with auditory feedback. Participants then completed a series of test blocks consisting of 40 trials each (without auditory feedback). Participants were not blindfolded, nor were they instructed to close their eyes during the test blocks. Previous studies have shown that tactile acuity improves when the participant views the tested hand [49]–[51]; therefore, we covered the participant's tested hand from view with a box (Figure 1) in order to avoid the possible confound that participants might perform better in the light – not because of differences between the light and dark conditions per se – but simply because they could view the back of their hand.

As in Experiment 1, participants were tested in the light (fluorescent overhead room lighting) and the pitch-dark (<0.01 lux). Unlike in Experiment 1, the investigator did not remain in the testing room with the participants. Therefore, participants in the dark were required to put on light-occluding goggles for a brief period (approximately 2–3 minutes) as the experimenter entered the room to initialize the equipment before each stimulation block. Except for these very brief periods, the participants were not blindfolded.

#### Experimental Design & Conditions

Participants were assigned to one of four groups in pseudorandom order. Participants in the non-deprived group completed 10 test blocks in the light and were never visually deprived. Participants in the other three (visually deprived) groups completed two test blocks before (in the light) – to obtain baseline tactile acuity – and three test blocks after (in the light) experiencing a period of visual deprivation. The sequence of events during the visual deprivation period (in the pitch-dark) differed by visual deprivation group (Figure 4).

To investigate whether short-term visual deprivation alone improves tactile spatial acuity, as reported [35], [36], we administered one test block after a 70-minute visual deprivation period to participants in the unstimulated group; these participants listened to music of their choice during the visual deprivation period.

To investigate whether and how tactile acuity changes over time with visual deprivation, we administered five test blocks during the visual deprivation period to participants in the repeatedly tested group.

To investigate whether unattended grating stimulation in the dark would improve tactile acuity, we administered four passive stimulation blocks followed by one test block during the visual deprivation period to participants in the passively stimulated group. These participants were instructed to ignore the grating stimuli contacting the finger during a passive stimulation block; during the passive stimulation, they listened to music of their choice. As in the test blocks, in each trial of a passive stimulation block the participant's tested finger was contacted with a grating twice, once oriented vertically and once horizontally (order chosen randomly). However, unlike during testing, the participant did not make any response (the computer program produced a sham response 700 ms after the end of stimulation, and the next trial therefore automatically commenced). The sequence of grating groove widths contacting the participant's finger in a passive stimulation block was the same sequence the participant experienced during the first or second test block, chosen randomly (if the participant had made a finger-movement error during the first or second test block, resulting in a discarded trial, the largest groove width in the stimulus set, 3.1mm, was given in its place during passive stimulation).

Participants took on average 7 minutes to complete a test or passive stimulation block; including set-up time by the experimenter, each block lasted approximately 9 minutes. Successive blocks were separated by 8-minute break periods during which participants were free to listen to music of their choice. For participants in the repeatedly tested and passively stimulated groups, the average elapsed time between the start of the initial block in the dark and the start of the final block in the dark was 68 minutes; participants in the unstimulated group sat in the dark for exactly 70 minutes before being tested. Participants in all three visually deprived groups remained in pitch-darkness during the break following the final testing block in the dark; these participants sat in a pitch-dark room for approximately 90 minutes (Figure 4).

### Experiment 3

#### Participants

Sixty-six normally sighted right hand-dominant students from McMaster University (35 men, 31 women, ages 18.1–25.7 years, median age 19.5 years) participated in Experiment 3. The same qualification criteria and handedness questionnaire used in Experiment 2 were used here.

#### Psychophysical Procedures

The psychophysical procedures were identical to those used in Experiment 2.

#### Experimental Design & Conditions

To ensure participant alertness, we recruited participants in sets of three and encouraged conversation during the visual deprivation period. Each set of three was assigned to one of two groups in alternating order: non-deprived and visually deprived.

Every participant was tested three times. The first test block served as a measure of the participant's baseline tactile acuity. This was followed by a 110-minute conversation period during which participants talked with one another or with an experimenter. The conversation period was followed by a second test block, after which the participant left the laboratory to take a 120-minute break. Following the break, the participant returned to the laboratory to complete a final test block. Participants took on average 8 minutes to complete a test block.

Non-deprived participants were always in the light. Visually deprived participants were in the pitch-dark during the conversation period and while completing the second test block. The visually deprived participants were in the pitch-dark for an average duration of 120 minutes.

The test blocks were administered in a testing room, and the conversation period took place in a separate conversation room. Participants in each set of three were tested sequentially (Figure S1). It was therefore inevitable that, as participants rotated into the different phases of the experiment, the first and third participant would at different times be alone in the conversation room. To maintain participant alertness during these periods, the participant in the conversation room conversed by remote two-way audio either with the experimenter or with a fellow participant who was waiting outside the laboratory.

### Data Analysis

We performed analyses of variance (ANOVA) with SPSS v19 (IBM Corp., Somers, NY) for Macintosh, with an alpha-level of 0.05. The dependent measure used in the statistical analysis of Experiment 1 was the participant's 70.71% correct threshold, obtained using a two-down one-up staircase procedure [47]. The dependent measure used in the statistical analyses of Experiments 2 and 3 was the mean of the posterior PDF of the participant's 76% correct threshold, obtained using a modified version of the ψ-method [39], [48].

## Supporting Information

Figure S1 **Sequence of events in Experiment 3.** (A–C) The participants were tested sequentially in the testing room, and then seated sequentially in the conversation room. (D) Each participant spent a total of 110-minutes in the conversation room. (E–G) The participants were then tested sequentially a second time. (H) All three participants left the laboratory for a 120-minute break and returned sequentially to be tested a final time (not shown). The image is not drawn to spatial or temporal scale.
(TIF)
