# Supplementary figures and images for: Short-Term Visual Deprivation Does Not Enhance Passive Tactile Spatial Acuity
Source: PLoS One. 2011 Sep 23;6(9):e25277. doi: 10.1371/journal.pone.0025277 (PMC3179498; doi:10.1371/journal.pone.0025277)

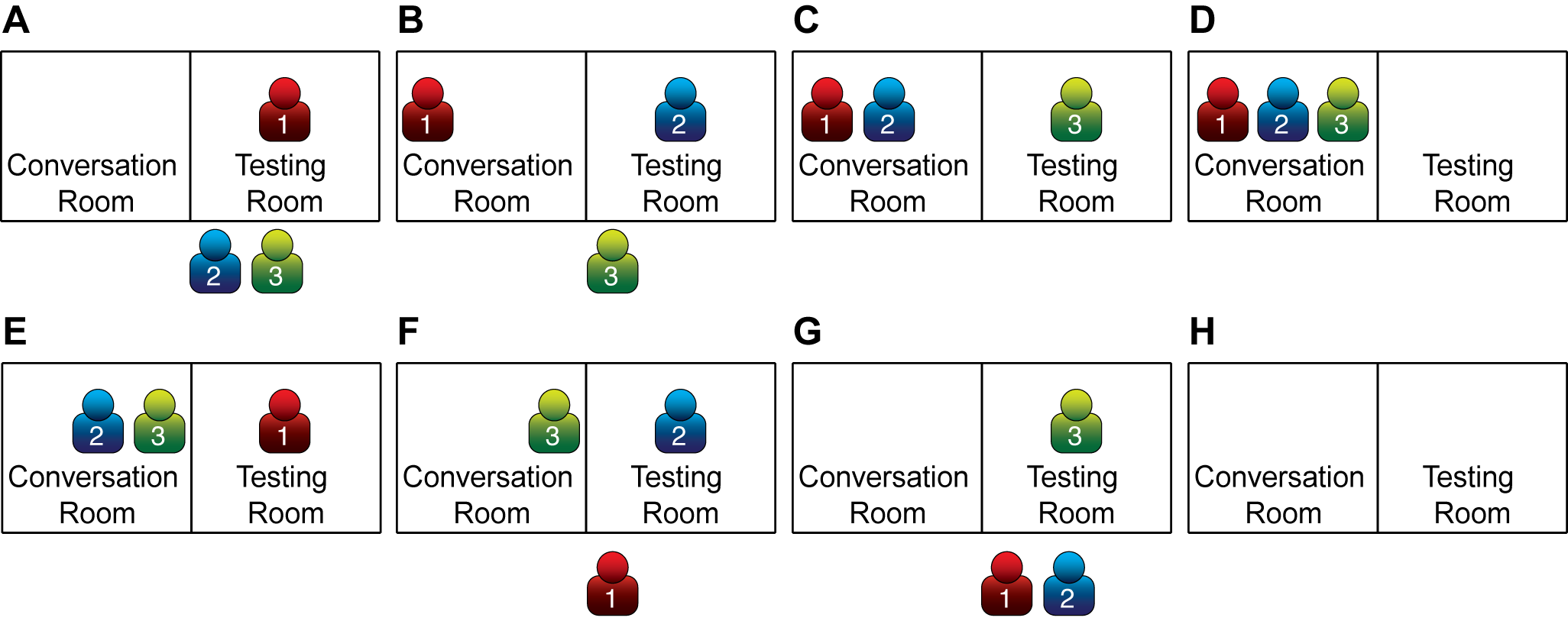

Supplement: Figure S1 — Sequence of events in Experiment 3. (A–C) The participants were tested sequentially in the testing room, and then seated sequentially in the conversation room. (D) Each participant spent a total of 110-minutes in the conversation room. (E–G) The participants were then tested sequentially a second time. (H) All three participants left the laboratory for a 120-minute break and returned sequentially to be tested a final time (not shown). The image is not drawn to spatial or temporal scale. (TIF) [file pone.0025277.s001.tif]
